# Phenotypic Plasticity in *Arabidopsis thaliana* Is Fitness‐Neutral, and Costs Are Lacking Across Experimental Environments

**DOI:** 10.1002/ece3.73427

**Published:** 2026-04-13

**Authors:** Maya L. Shamsid‐Deen, Kenneth D. Whitney

**Affiliations:** ^1^ Department of Biology University of New Mexico Albuquerque New Mexico USA

**Keywords:** *Arabidopsis thaliana*, fitness neutral, non‐adaptive plasticity, phenotypic plasticity, plasticity evolution

## Abstract

Phenotypic plasticity produces multiple phenotypes depending on the environment, and its evolutionary trajectory is determined by the fitness consequences of those phenotypes. Adaptive plasticity in traits would reduce plasticity in fitness across the range of environments experienced by a species. However, it is unclear how often plasticity is adaptive, neutral, or maladaptive. Here, we quantified plasticity in *Arabidopsis thaliana* across multiple environments, asking whether accessions with high plasticity across eight traits have low plasticity in seed output. In addition, we examine if phenotypic plasticity is adaptive, neutral, or maladaptive and test for costs of plasticity. We found no significant relationships between trait plasticity and either seed output or plasticity in seed output, indicating that plasticity is neutral for the traits and environments examined. We also found no evidence of costs of plasticity. We found that plants that exhibit plasticity in the number of rosette leaves when fertilized had higher seed output under control conditions. Given that phenotypic plasticity in the number of rosette leaves is present in accessions with high seed output, phenotypic plasticity in this trait may be affiliated with other traits under strong selection rather than under direct selection itself. Results of this study contribute to our understanding of the frequency of neutral plasticity.

## Graphical abstract

Phenotypic plasticity allows organisms to produce different phenotypes across environments, but its fitness consequences determine whether it is adaptive, neutral, or maladaptive. In *Arabidopsis thaliana* , we found no significant relationship between trait plasticity and fitness, suggesting plasticity is largely neutral and without detectable costs for the traits and environments examined.

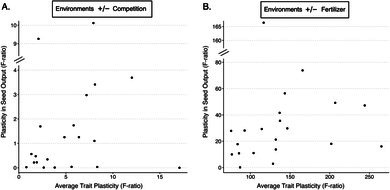

## 1 Introduction

Phenotypic plasticity, or the expression of different phenotypes based on the environment, can provide raw material on which natural selection can act. Incorporating phenotypic plasticity into evolutionary theory is essential, as models assuming a one‐to‐one genotype to phenotype relationship are incomplete (Scheiner 1993). Selection on phenotypic plasticity produces a diversity of evolutionary outcomes, provided that plasticity is heritable (Scheiner and Lyman 1989; Hendry 2016). Plastic responses may be maintained, refined, reduced, or become genetically fixed, depending on whether they enhance, diminish, or have neutral fitness effects (Lee et al. 2022). Consequently, understanding the evolutionary trajectory of phenotypic plasticity requires an understanding of how plastic responses impact fitness.

The widespread occurrence of phenotypic plasticity has prompted extensive research into its adaptive significance, given that its prevalence may be driven by fitness benefits. Plasticity is adaptive when fitness is maintained across different environments (low plasticity in fitness) or when plastic genotypes exhibit increased fitness across environments relative to non‐plastic genotypes (Alpert and Simms 2002). When immediate trait responses to the environment increase fitness, such as in heterogeneous environments (West‐Eberhard 2003) and during the initial colonization of a novel environment (Lande 2015; Donohue et al. 2001), increased phenotypic plasticity could be selected for. A meta‐analysis of 31 reciprocal transplant studies found that adaptive plasticity was more common than non‐adaptive plasticity, with 69% of plastic responses being adaptive (Palacio‐López et al. 2015). However, in this study, “adaptive” was not defined as fitness benefit per se but was inferred based on comparisons of phenotypes to those of resident populations assumed to represent local fitness optima (Palacio‐López et al. 2015), perhaps limiting interpretability. Strong evidence supports the idea that plasticity in certain traits can be adaptive in some plant species. Studies have linked plasticity in shade tolerance and herbivore defense to increased bud, flower, and fruit production (Schmitt et al. 1995), and enhanced resistance to herbivores and pathogens (Agrawal et al. 1999; Karban et al. 1999). In other hypothesized adaptive plastic responses, such as plasticity in root secondary growth and length, a direct fitness benefit has yet to be demonstrated in multiple environments (Schneider and Lynch 2020; Grossman and Rice 2012).

Maladaptive plasticity, which is sometimes referred to as non‐adaptive plasticity, produces plastic responses that are farther from a local optimum or that result in reduced fitness (Ghalambor et al. 2007). Maladaptive plasticity often occurs in response to environmental stress (van Kleunen and Fischer 2005; Ghalambor et al. 2007). Stressors such as extreme pH or temperature may disrupt development and/or physiological processes, leading to increased phenotypic variance. Further, low resources inhibit organisms from growing larger or producing as many offspring as possible, thus reducing their fitness (van Kleunen and Fischer 2005). Additional cases of maladaptive plasticity may include unreliable environmental cues that elicit phenotypes mismatched with the environment, lag times in phenotypic responses, developmental range limits, and constraints on future plastic responses due to previous plastic responses, that is, the plasticity‐history limit (van Kleunen and Fischer 2005; DeWitt et al. 1998). Lastly, maladaptive plasticity could also be due to developmental instability or from genetic trade‐offs, including pleiotropic effects and genetic correlations in which alleles underlying plastic responses reduce fitness through their effects on other traits (Abley et al. 2016; van Kleunen and Fischer 2005; DeWitt et al. 1998). Ultimately, selection against maladaptive phenotypic plasticity may result in genetic assimilation, where an initially plastic trait becomes genetically fixed (Waddington 1953).

Although infrequently discussed (but see Acasuso‐Rivero et al. 2019), phenotypic plasticity can be fitness‐neutral. Neutral phenotypic plasticity may result from drift or genetic correlations with other traits under selection rather than positive selection on plasticity itself (van Kleunen and Fischer 2005). For example, in *Oryza sativa* L., genomic regions associated with phenotypic plasticity in panicle weight in response to CO_2_ have pleiotropic effects on biomass and other fitness‐related traits (Kikuchi et al. 2017). In *Arabidopsis thaliana* , Pigliucci and Schmitt (1999) found that photomorphogenic genes had pleiotropic effects on the plasticity of multiple traits such as bolting time and number of basal branches. Epistatic interactions may further obscure phenotypic plasticity's relationship with fitness by introducing environment‐specific effects that are difficult to detect (Des Marais et al. 2013). Pleiotropy and epistatic interactions may explain the persistence of plasticity in the absence of a direct fitness benefit. However, a comprehensive understanding of the frequency of neutral phenotypic plasticity across species and traits has yet to be developed.

Importantly, classifications of plasticity as adaptive, maladaptive, or neutral describe fitness consequences in a given context, but these states are not evolutionarily fixed and may shift under changing environmental conditions and selection pressures. For example, maladaptive plasticity can occur when organisms encounter a new environmental cue, as natural selection has not yet acted on the underlying epigenetic or genetic controls of plasticity (Grether 2005). Under selection, these responses can be diminished or refined. Recent work has shown that initial plastic responses that were further away from a local optimum were under stronger selection than adaptive plasticity and drove rapid adaptations in gene expression (Ghalambor et al. 2015). When plasticity reduces relative fitness, directional selection can promote the evolution of compensatory genetic or epigenetic changes (Grether 2005). However, empirical evidence for rapid adaptation via non‐adaptive phenotypic plasticity remains limited. Additionally, the fitness effects of phenotypic plasticity may be context‐dependent, particularly when genes affect traits under opposing selective pressures across environments (Valladares et al. 2007).

Phenotypic plasticity may exhibit costs that are associated with the maintenance of sensory and regulatory mechanisms of plasticity (DeWitt et al. 1998). Costs associated with phenotypic plasticity have been theorized to constrain its evolution and may help explain why adaptive plasticity is not ubiquitous (Ernande and Dieckmann 2004; Auld et al. 2010). While costs have been difficult to demonstrate and are often minimal, they have been shown to be more pronounced under stressful conditions (van Kleunen et al. 2000; van Buskirk and Steiner 2009). In a meta‐analysis of 536 selection estimates, costs of plasticity were no more frequent than costs of canalization (van Buskirk and Steiner 2009). This conundrum of missing costs may be due to a common analytical approach for identifying costs of plasticity. Tests of selection on plasticity incorporate the benefits of plasticity, which may offset costs, making the latter undetectable (Hendry 2016; de Lisle and Rowe 2023).

Plants are well‐suited for investigating the relationship between fitness and phenotypic plasticity (van Kleunen and Fischer 2005). As sessile organisms, they are restricted to their location of germination and are forced to cope with changes in their environment, which may further help explain the ubiquity of phenotypic plasticity identified in plant systems (Palacio‐López et al. 2015; Acasuso‐Rivero et al. 2019). Using the model plant *A. thaliana* , we aimed to elucidate plasticity's relationship with fitness across gradients of competition, nutrients, and herbivory. Prior research in *A. thaliana* has demonstrated that biotic conditions (specifically competition) can modify abiotic selection and influence plastic responses along with adaptive strategies (Lorts and Lasky 2020). We varied abiotic and biotic conditions simultaneously to mirror conditions under rapid environmental change, where multiple abiotic and biotic factors change simultaneously (Kawecki et al. 2012). We define phenotypic plasticity as environment‐dependent variation in trait expression. For our first aim (A1), we investigated the relationship between phenotypic plasticity in traits and plasticity in fitness. We hypothesized that, if plasticity is adaptive, plants can optimize their phenotypes for the environment, leading to stable fitness across conditions (i.e., low plasticity in seed output). Our alternative hypothesis posited that genotypes that are highly plastic in traits also have high plasticity in seed output. This alternative could arise from the costs or limits of plasticity or through an ability to capitalize on plentiful resources to exhibit higher fitness. Secondly (A2), we aimed to identify whether plasticity was adaptive, neutral, or maladaptive for our set of traits and environments, by examining the slope of the relationship between trait plasticity and absolute fitness. Finally, for aim three (A3), we sought to identify any costs associated with phenotypic plasticity using the regression approach of Scheiner and Berrigan (1998), which tests for detriments to fitness for plastic traits relative to fixed traits in a specific environment.

## 2 Materials and Methods

### 2.1 Plant Material

*Arabidopsis thaliana* (L.) Heynh (Brassicaceae) is a highly self‐fertilizing annual plant that has been shown to be phenotypically plastic in germination timing (Donohue et al. 2005), shade avoidance responses (Pigliucci et al. 1999; Dorn et al. 2000), shoot branching (de Jong et al. 2019), and the total number of rosette leaves (Samis et al. 2019). We selected 21 accessions throughout *A. thaliana* 's global range (Table 1) to compare phenotypic plasticity in traits. These accessions were obtained as single‐seed descent lines from the Arabidopsis Biological Resource Center (ABRC) and thus represent distinct genotypes.

**TABLE 1:** Study accessions with ARBC CS stock number, name, collection location, and identified admixture group from 1135 genomes project (Alonso‐Blanco et al. 2016).

CS number	Name	Location	Genetic cluster
CS76433	Altai‐5	Xinjiang, China	Central Asia
CS77101	MNF‐Riv‐21	Michigan, USA	Central Asia
CS76496	Gr‐1	Graz, Austria	Central European
CS76609	Tac‐0	Washington, USA	Central European
CS76459	Ca‐0	Camberg, Germany	Germany
CS79014	FM‐11	New York, USA	Germany
CS77124	NC‐6	North Carolina USA	Germany
CS76633	Yo‐0	California, USA	Germany
CS76367	Lago‐1	Rocigliano‐Lago, Italy	Italy, Balkans, Caucasus
CS76826	Eden‐1	Eden, Sweden	Northern Sweden
CS76649	Aitba‐1	Ait Barka, Morocco	Relict
CS1064	Can‐0	Las Palmas, Canary Islands	Relict
CS76789	Cvi‐0	Cape Verde	Relict
CS76943	IP‐Her‐12	Herguijuela, Spain	Relict
CS76943	IP‐Hum‐2	Humienta, Spain	Relict
CS77169	IP‐Per‐0	Perin, Spain	Relict
CS78844	IP‐Vim‐0	Villarino de Manzanas, Spain	Relict
CS75719	Tanz‐1	Ketumbeine Forest, Tanzania	Relict
CS76790	CYR	Maine‐et‐Loire, France	Western European
CS76527	Kin‐0	Michigan, USA	Western European
CS76576	Pog‐0	British Columbia, Canada	Western European

### 2.2 Seed Bulking

We grew 12 plants from seed from each of the 21 accessions in the University of New Mexico Biology greenhouses to accumulate enough seeds for the plasticity experiment (below) and to reduce any possible maternal effects (Kawecki et al. 2012). All accessions were germinated in October 2020 and grown through a full life cycle, with senescence completed by March 2021. Each plant was grown individually in a separate seed‐starting pot insert (one plant per cell) within a 52‐cell tray, with each pot tagged for identification. Plants were grown in Sungro Horticulture Metro Mix (Agawam, MA, USA) 360 soil. This soil was treated with Marathon 1G (Hummert International), a granular insecticide, and a slow‐release fertilizer Osmocote 14 N–14 P–14 K was added per the planting guidelines used by the ARBC (Rivero et al. 2014). All seeds were vernalized on the soil surface for 1 week at 3.8°C before being placed in the greenhouse. Plants were misted daily or were bottom watered (in the event daily misting could not occur) to be given continuous access to water until all plants had germinated. Following germination, trays were bottom watered three times a week. We rotated the trays 180° after each watering to reduce microclimate effects. Plants were grown under supplemental light (16 h a day; BML Spydr 600 LED Grow Light Grow‐Max Spectrum—120v, Austin, TX, USA) while the greenhouse temperature was set to 23°C. Plants were allowed to self‐fertilize and bulk seed was collected using the Arasystem (Betatech BVBA, GER), a seed‐starting pot and tray system of tubes and cups that prevent cross‐pollination, facilitate seed collection, and prevent mixing of seeds from different plants.

### 2.3 Experimental Design

To initiate our experiment, plants were grown in Sungro Horticulture Metro Mix 820 soil treated with Marathon 1G insecticide. Sungro Horticulture Metro Mix 820 was selected as the soil given that 360 was discontinued, but it is comparable to 360. All plants were planted in trays of 36, 4.93 cm × 5.66 cm × 5.66 cm cell length × width × depth, pots from Greenhouse Megastore (Danville, Illinois, USA). Seeds were germinated following the same procedures as seed bulking. Following germination, trays were bottom watered two to three times a week, and we alternated the trays from the north to the south end of the bench before each watering. Plants were grown under the same lighting conditions as detailed above. The greenhouse temperature was set to a maximum of 21°C and a minimum temperature of 10°C. To elicit plastic responses, we implemented a full‐factorial experimental design manipulating three factors, each at two levels: competition (+/−), fertilization (+/−), and herbivory (+/−), yielding eight treatment combinations. Six plants per treatment combination from each of the 21 accessions produced a sample size of 1008 plants.

The competition treatment was initiated immediately after vernalization. We selected *Lolium multiflorum* Lam. (annual ryegrass) as the competitor, as it is native to the same region as *A. thaliana* and has been widely introduced as a cover crop (Roché et al. 2007). In two opposite corners of every pot assigned to the (+) level of the competition treatment, two seeds of *L. multiflorum* were planted as the competitors. Seeds were sourced from Papaws Garden Supply, L.L.C. (Seymour, Indiana, USA). All seeds successfully germinated, and all *L. multiflorum* plants persisted through the experiment. The fertilizer treatment was applied weekly beginning 12 days after vernalization when all plants had germinated and had four leaves. One tablespoon or approximately 16.1 g of Peters Professional 20 N–20 P–20 K General Purpose Fertilizer was dissolved per gallon to produce the solution. A Corning Stripettor Ultra Pipet Controller (Corning Inc., Arizona, USA) was used to apply 25 mL of fertilizer solution. Plants that were assigned to the (−) level of the fertilizer treatment received 25 mL of water to control for the fertilizer solution plants received in the (+) level. The herbivory treatment was applied when plants reached 5.5 cm in rosette diameter or if plants remained < 5.5 cm, when there was the development of an inflorescence bud. *Trichoplusia ni (Hüber)* (cabbage looper), known to feed on *Arabidopsis* and complete its life cycle on this plant (Jander et al. 2001), was selected as the herbivore. Second instar caterpillars were purchased from Benzon Research, Incorporated (Dearborn, Michigan, USA). Caterpillars were fed Benzon's proprietary artificial diet prior to use in the experiment. A single cabbage looper larva was applied to a single rosette leaf using insect clip cages made from plastic Petri dishes that measured 35 mm in diameter × 12 mm in height (NEST Scientific, Woodbridge, New Jersey, USA). Caterpillars were allowed to feed until the leaf was completely eaten. Each plant assigned to the (+) herbivory treatment had one leaf removed via this method.

### 2.4 Trait Measurements

Eleven traits known to be plastic in *A. thaliana* (Pigliucci et al. 1999; Dorn et al. 2000; Samis et al. 2019) and/or are relevant to fitness were measured (Palacio‐López et al. 2020). We counted the *number of rosette leaves at 1 month*. We lifted all top leaves to count any underlying rosette leaves. *Rosette diameters* at 1 and 2 months were measured using a ruler from the widest leaf‐tip–to–leaf‐tip span to the nearest 0.1 cm. We recorded *days vegetative* by taking the difference between the germination day and the surveyed flowering date (censused daily excluding 1 day on the weekends). Flowering was defined as the corolla being fully visible and the *days flowering* were the total number of days from initiation of flowering to flower senescence of the last flower. We counted the *number of inflorescence branches* at harvest and only included inflorescence branches that grew from the base of the rosette. Axillary branching was not recorded. We measured the *maximum plant height* from the soil to the tallest end of the inflorescence when stretched upright to its fullest extent. Measurements were recorded to the nearest millimeter using a meterstick. Silique counts were made monthly once siliques started to develop in an effort to capture the peak silique production. Because siliques were produced and senesced over time, counts were not summed across months (which would have double‐ or triple‐counted the same individual silique); rather, the maximum number of silique*s* recorded for each plant during silique production was utilized for analyses as our *silique count*. It is a conservative estimate of the total number of siliques produced because it captures the total number produced minus a small number that had senesced and fallen off by the peak date. In addition, we harvested three siliques from each plant at first dehiscence. These siliques were dissected, and seed numbers were recorded per silique. The average seed number of the three siliques represents our *average seed number per fruit*, which was multiplied by the maximum number of siliques per plant to produce an estimate of plant‐level seed production. These estimates were then averaged across all plants in an accession to produce the accession‐level *average seed output*. *Longevity* was the total number of days from germination to plant senescence.

### 2.5 Computation of Phenotypic Plasticity Dataset

We first excluded the silique count and the average seed number per fruit as these were used to calculate the total seed number. We generated *F*‐ratios from factorial ANOVAs with type II sums of squares representing the environment effect on our trait responses. We utilized *F*‐ratios to quantify phenotypic plasticity in each of our traits. We employed *F*‐ratios as they provide a robust, unitless metric of the strength of phenotypic responses across treatment levels (i.e., groups) relative to within‐treatment variability, making them suitable for cross‐trait comparisons (Schlichting 1986). *F*‐ratios greater than one indicate the variation in a trait's response is greater between the environments (groups) relative to the variation within a single environment and thus indicates phenotypic plasticity. *F*‐ratios that are less than or equal to one demonstrate the absence of phenotypic plasticity to an environment. *F*‐ratios that are equal to one indicate there was no difference in the trait variation between environments relative to within an environment. Finally, *F*‐ratios less than one indicate that the variation within an environment is greater than the variation between environments. Here, the environment does not explain the variation in trait responses. Unlike coefficients of variation (CV), which cannot be reliably compared across traits or environments even when mean‐scaled (Pélabon et al. 2020), the *F*‐ratios quantify the strength of environmental effects relative to residual variance, allowing comparisons of plastic responses across traits analyzed using identical models. This approach allowed us to identify which treatments elicited plastic responses in our multi‐factorial experimental design.

To select which environments would serve as predictors for the ANOVA model, we used Akaike information criterion (AICc) that corrects for small sample sizes from the package AICcmodavg (Mazerolle 2023; Burnham and Anderson 2002) to compare individual models that included the effects of treatments and their interactions for each trait in R version 4.0.3 (R Core Team 2023). We identified the following model as the best fit for our trait data: *Trait ~ Fertilizer × Competition*, which includes the additive effect of fertilizer, the additive effect of competition, and their interaction. Preliminary model testing indicated that the herbivory treatment did not influence the expression of the phenotypic traits; therefore, we excluded this treatment from subsequent analyses. *F‐*ratios were calculated separately for each accession, for each of the nine traits, and for the two relevant treatment gradients using a type II ANOVA. Type II sums of squares were used because the design was not full rank, and our focus was on estimating the main effects of competition and fertilizer while accounting for their interaction term. For each accession, trait plasticity was quantified as the *F*‐ratios associated with the environmental effect from identical linear models. We then averaged these *F*‐ratios across traits to obtain an accession‐level estimate of overall plasticity. Because all traits were analyzed using identical models, *F*‐ratios were directly comparable and were averaged using the arithmetic mean without weighting. Plasticity in seed output was excluded because seed output was analyzed as the response variable in A1. One accession (Yo‐0) was excluded from the fertilizer dataset due to low to no seed production for most plants. These computations yielded a dataset of 378 *F*‐ratios.

### 2.6 Phenotypic Plasticity and Fitness Analysis

To evaluate A1, we constructed linear, cubic, and quadratic models to test whether average trait plasticity predicted plasticity in seed output. We compared our models using AICc‐based model selection (Table A1). For each environmental gradient (fertilizer +/− and competition +/−), the best‐supported model form was used to assess the relationship between average trait plasticity and seed output plasticity. Model assumptions of normally distributed residuals and homoscedasticity were reviewed visually using a histogram and a residuals versus fitted values plot, as well as statistically through the Shapiro–Wilk test. For the competition environments, the *F*‐ratios were logarithmically transformed to meet the assumptions of homoscedasticity and normality (Steel and Torrie 1980). Model assumptions could not be met for the fertilizer environment. Given these model violations and the positive right skew of our response variable, we employed a generalized linear model with a Gamma error distribution and log link. To assess the general relationship between trait plasticity and absolute seed output (A2), we regressed seed output on average trait plasticity calculated from all traits to identify their relationship under each of the environmental gradients. Using an AICc‐based model selection, we compared linear, cubic, and quadratic models (Table A2). The model assumption of normality was tested using the Shapiro–Wilk test. We log‐transformed our response variable in the competition environment analysis and square‐root transformed seed output in the fertilizer environment analysis. To visualize results, all figures were generated in R using the ggplot2 package (Wickham 2016).

### 2.7 Correlations in Trait Plasticities

To investigate if phenotypic plasticity is correlated across traits under our environments (competition +/− and fertilizer +/−), we estimated Spearman's *r* rank correlation matrices using the R package, Hmisc version 5.2‐3 (Harrell 2025). In addition to trait‐to‐trait correlations, we also included the average trait plasticity to examine whether plasticity in individual traits is correlated with overall average plasticity. We tested for statistical significance using an alpha level of 0.05 and applied the Bonferroni correction given our multiple comparisons.

### 2.8 Phenotypic Plasticity Cost Analysis

Costs of phenotypic plasticity are exhibited when canalized accessions have higher than expected fitness and plastic accessions have lower than expected fitness or when more plastic accessions exhibit lower fitness than less plastic accessions (DeWitt et al. 1998). To account for trait values and isolate costs specifically due to plasticity (A3), we applied a regression model developed by Scheiner and Berrigan (1998) for each environment (*e*):

$$\overset{-}{W_{e}}=\beta_{1}+\beta_{2}\overset{-}{X_{e}}+\beta_{3}\overset{-}{plX_{e}}+\text{Error}$$

where $\bar{W}$ is the average seed output per accession in a given environment (control, competition, and fertilizer). Per accession, $\bar{X}$ is the mean trait value in a given environment. Per accession, $\bar{\mathrm{plX}}$ represents the mean plasticity of that trait, quantified as the *F*‐ratio for the environmental effect across the competition +/− and fertilizer +/− gradients. The betas are partial regression coefficients. Positive and statistically significant $\beta_{2}$ values indicate that increases in a trait value correspond to increased seed output, and a negative relationship is exhibited if $\beta_{2}$ values are negative and statistically significant. The plasticity coefficient, $\beta_{3}$, that is negative and statistically significant, indicates costs of plasticity, while a statistically significant positive $\beta_{3}$ coefficient indicates a cost for canalization in the competition or fertilizer environments (Scheiner and Berrigan 1998). Under control conditions, we tested whether plasticity to competition or fertilizer influenced seed output when those environmental cues were absent. Identifying such associations would suggest potential maintenance costs of plasticity should $\beta_{3}$ be negative and statistically significant. Positive and significant $\beta_{3}$ in the control environment indicate an association with high seed output. We standardized the coefficients using the *lm.beta* package to standard deviation units to compare across traits and identify whether the trait value or plasticity in that trait had a greater effect on seed output. We corrected *p* values for running eight regressions (one for each trait) per environment via the Bonferroni correction.

## 3 Results

Our trait measurement dataset was derived from 902 plants representing 21 accessions that survived the entirety of the experiment. A dataset of 378 plasticity measurements (*F*‐ratios) was generated from these trait measurements. The final accession level dataset comprised the following averages: 42 measurements of trait plasticity, 41 measurements of seed output, and 42 measurements of plasticity in seed output.

**FIGURE 1:**
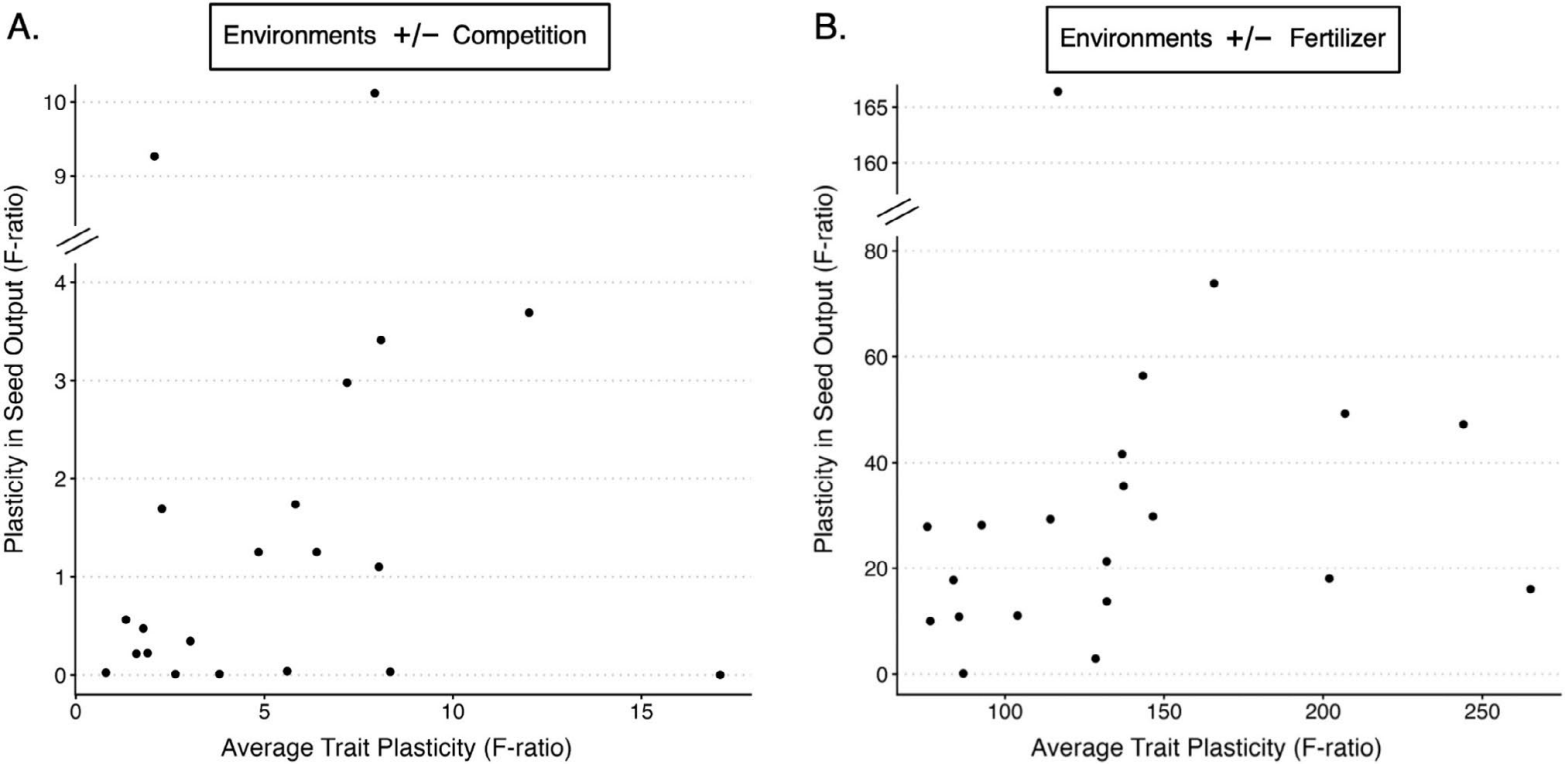
Scatterplots depicting the average trait plasticity across eight traits versus plasticity in seed output. Points represent the average *F*‐ratios calculated for each accession. (A) Depicts competition environments, while (B) depicts fertilizer environments. The *y*‐axis is truncated at the diagonal axis breaks for visualization.

**FIGURE 2:**
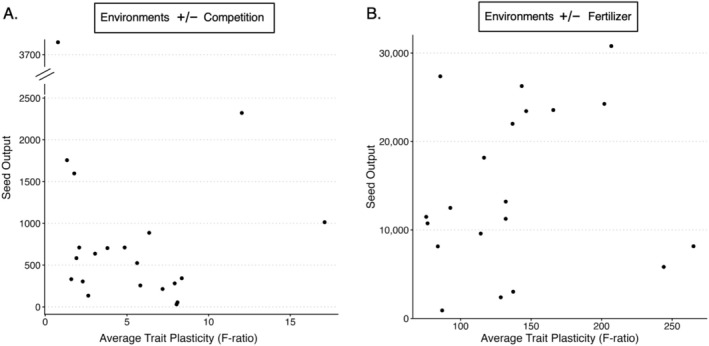
Scatterplots depicting the average trait plasticity across eight traits versus seed output. Points are individual accession averages. (A) Depicts competition environments, while (B) depicts fertilizer environments. The *y*‐axis in panel (A) is truncated at the diagonal axis breaks for visualization.

In our investigation of phenotypic plasticity's relationship with plasticity in fitness (A1), we did not find a statistically significant relationship between average trait plasticity and plasticity in seed output in the competition environments (*β* = −0.054, 95% CI: −0.339 to 0.231, *t* = −0.396, *p* = 0.696; Figure 1; Table A3). Under fertilizer environments, average trait plasticity did not predict plasticity in seed output (*β* = 0.003, 95% CI: −0.007 to 0.016, *t* = 0.713, *p* = 0.485; Figure 1; Table A4). Although the quadratic model had a lower AICc than the linear model (Table A2), the improvement in fit was modest relative to the increase in model complexity. Given the limited sample size, we retained the more parsimonious linear model for inference. For the relationship of plasticity with absolute seed output (A2), average trait plasticity in response to competition did not predict seed output (*β* = −0.041, 95% CI: −0.177 to 0.095, *t* = −0.636, *p* = 0.532), nor did plasticity in response to fertilizer (*β* = 0.110, 95% CI: −0.264 to 0.485, *t* = 0.619, *p* = 0.544; Figure 2; Table A5).

**FIGURE A1:**
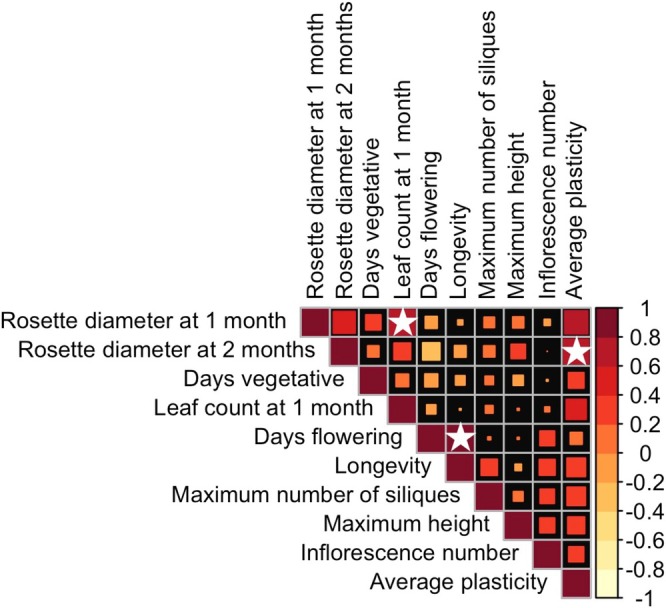
Spearman rank correlation matrix of trait plasticity coefficients under competition. Color and square size represent the direction and magnitude of Spearman's *ρ* (range −1 to 1). White stars denote significant correlations (*p* < 0.05).

**FIGURE A2:**
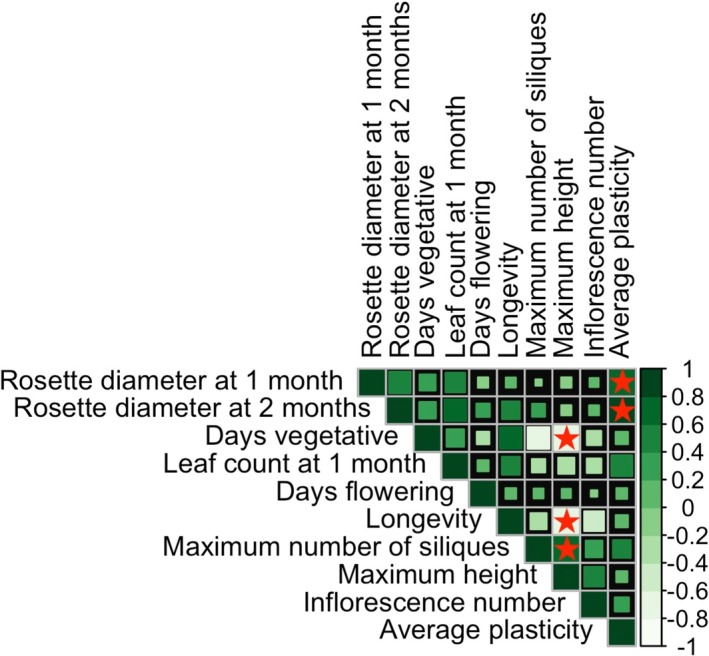
Spearman rank correlation matrix of trait plasticity coefficients under fertilizer. Color and square size represent the direction and magnitude of Spearman's *ρ* (range −1 to 1). Red stars denote significant correlations (*p* < 0.05).

We examined whether average plasticity represented plasticity of individual traits and found that it was: correlation coefficients (*r_s_*) were always positive (range 0.16–0.71, mean *r*_s_= 0.39 in the competition environments, range 0.15–0.91, mean *r*_s_= 0.39 in the fertilizer environments). Phenotypic plasticity was largely uncorrelated across traits. In the competition environments, we found that the plasticity in days flowering was positively correlated with plasticity in longevity (*r*_s_= 0.81, *p* < 0.001) while plasticity in rosette diameter measured at 1 month was positively correlated with plasticity in the number of leaves at 1 month (*r_s_* = 0.73, *p* = 0.006). Under the fertilizer environments, plasticity in maximum plant height was significantly negatively correlated with plasticity in two traits: days vegetative (*r*_s_= −0.73, *p* = 0.006) and longevity (*r_s_* = −0.77, *p* = 0.002). Plasticity in maximum plant height was positively correlated with plasticity in the maximum number of siliques (*r_s_* = 0.78, *p* = 0.001). All other correlations were nonsignificant (Tables A6 and A7; Figures A1 and A2).

**FIGURE 3:**
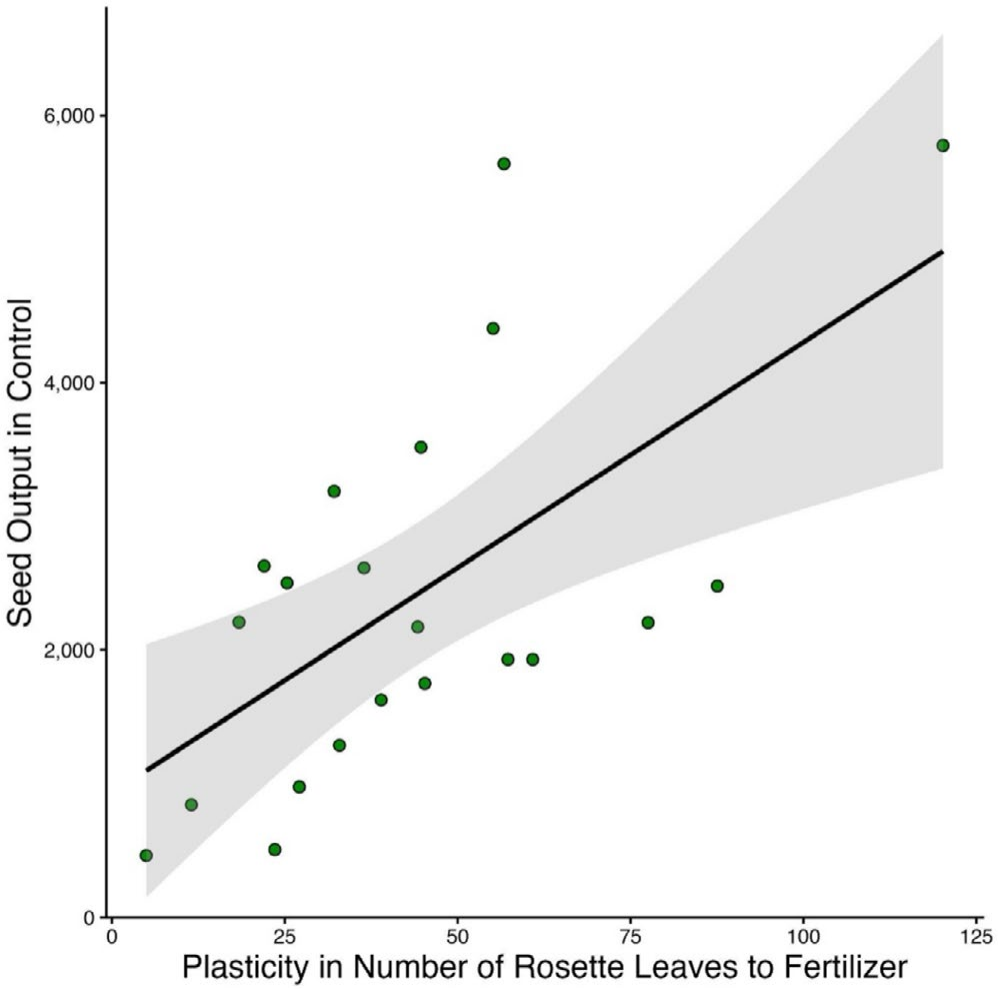
Scatterplot depicting plasticity in the number of rosette leaves at 1 month old in response to fertilizer versus seed output in the control environment. Points are individual accession averages, while shading represents 95% confidence intervals. The fitted line represents the Scheiner and Berrigan (1998) regression model. The relationship was positive and statistically significant after Bonferroni correction (adjusted *p* = 0.01).

We did not find a cost to plasticity in any of the measured traits (A3; Table 2). Plasticity in the number of rosette leaves at 1 month old in response to fertilizer was positively associated with higher seed output in the control environment (adjusted *p* = 0.01; Figure 3; Table 3).

**TABLE 2:** Summary of regression analysis of seed output on trait values and plasticity in the competition (left) and fertilizer (right) environments. Trait value coefficients and plasticity coefficients are standardized to a one‐unit standard deviation. Significant Bonferroni‐adjusted *p* values are illustrated via stars with **p < 0.01. Negative coefficients are associated with a reduction in seed output, while positive coefficients are associated with an increase. A significant negative coefficient for plasticity would indicate a cost of plasticity, and a significant positive coefficient for plasticity would indicate a cost of being canalized.

Trait	Competition environment			Fertilizer environment		
	Trait coefficient (*β*)	Plasticity coefficient (*β*)	*R* ^2^	Trait coefficient (*β*)	Plasticity coefficient (*β*)	*R* ^2^
Number of rosette leaves	0.362	−0.066	0.14	0.302	0.186	0.16
Rosette diameter at 1 month	0.229	−0.095	0.05	0.063	0.145	0.04
Rosette diameter at 2 months	0.407	0.186	0.19	0.156	0.007	0.02
Days vegetative	0.509	0.142	0.30	−0.669	0.091	0.39
Maximum height	0.721**	−0.046	0.54	0.544	0.217	0.39
Inflorescence number	0.716**	−0.063	0.53	0.009	0.156	0.03
Days flowering	−0.176	−0.419	0.18	0.711**	0.124	0.50
Longevity	0.716	0.022	0.50	0.324	−0.150	0.14

**TABLE 3:** Summary of regression analysis of seed output on trait values and plasticity in the control environment. Plasticity in a trait in response to competition's effect on seed output in the control environment is illustrated on the left, while plasticity to fertilizer is on the right. Trait value coefficients and plasticity coefficients are standardized to a one‐unit standard deviation. Significant Bonferroni‐adjusted *p* values are illustrated via stars with *p < 0.05 and **p < 0.01. Negative coefficients indicate a reduction in seed output while positive coefficients indicate an increase. A significant negative coefficient for plasticity indicates a cost for the maintenance of phenotypic plasticity, and a significant positive coefficient for plasticity indicates an association with high seed output.

Control environment	Plasticity to competition			Plasticity to fertilizer		
Trait	Trait coefficient (*β*)	Plasticity coefficient (*β*)	*R* ^2^	Trait coefficient (*β*)	Plasticity coefficient (*β*)	*R* ^2^
Number of rosette leaves	0.453	0.104	0.21	0.373	0.577*	0.53
Rosette diameter at 1 month	−0.169	0.259	0.04	−0.120	0.508	0.22
Rosette diameter at 2 months	0.628*	0.130	0.47	0.673*	0.013	0.46
Days vegetative	0.527	0.236	0.37	0.653*	−0.153	0.33
Maximum height	0.737**	−0.047	0.56	0.984**	0.327	0.61
Inflorescence Number	0.341	−0.286	0.14	0.172	−0.240	0.12
Days flowering	−0.194	−0.296	0.15	−0.258	0.006	0.07
Longevity	0.647**	−0.215	0.50	0.511	0.234	0.48

## 4 Discussion

### 4.1 Phenotypic Plasticity Is Fitness‐Neutral

Our study revealed no significant relationships between trait plasticity and plasticity in seed output, nor between trait plasticity and seed output. This suggests that, for our set of traits and environments, plasticity is neither adaptive nor maladaptive but is instead neutral. Our results align with other studies that question the frequency at which phenotypic plasticity is adaptive (Acasuso‐Rivero et al. 2019). Neutral phenotypic plasticity may be more common than previously thought. Prior research in plants has demonstrated the ubiquity of plasticity, with almost half of the traits examined shown to be plastic (Palacio‐López et al. 2015). It was noted that there was a surprisingly high prevalence of non‐adaptive plasticity, and the authors hypothesized this may be a form of bet hedging, where phenotypic variance is favored in the presence of unreliable environmental cues or extensive environmental heterogeneity (Scheiner et al. 2012; Scheiner 2014). Consistent with this idea, Abley et al. (2016) discuss that in unpredictable environments lacking reliable cues, selection may favor increased within‐environment phenotypic variance rather than a single plastic response that could prove maladaptive. Theory posits that neutral and maladaptive plasticity manifests in novel environments because the associated selection pressures have not acted to refine phenotypic responses (Ghalambor et al. 2007; Price et al. 2003). However, a meta‐analysis across diverse organisms, traits, and global environments suggested that neutral and, potentially, maladaptive plasticity may be more common in native environments than previously thought, calling into question whether non‐adaptive plasticity only emerges in novel conditions (Acasuso‐Rivero et al. 2019).

### 4.2 Phenotypic Plasticity May Persist Through Genetic Associations

The persistence of plasticity even in the absence of fitness benefits may be due to a pleiotropic genetic association with other traits under selection. Our results demonstrated that accessions with high plasticity in rosette leaf number in response to fertilizer also showed higher seed output in the control environment (Figure 3), indicating an association of plasticity and high fitness, perhaps through linkage disequilibrium or unlinked modifier alleles (de Lisle and Rowe 2023). Further work is needed to determine whether phenotypic plasticity is directly associated with traits under selection or is an indirect byproduct of the underlying genetic architecture of the organism.

### 4.3 The Good Condition of an Organism or the Environment May Mask Costs

We did not detect costs of phenotypic plasticity under either competition or fertilizer treatments. While theoretical work predicts that costs should be widespread, empirical studies often fail to detect them or report only minimal effects. Of 207 analyses, only 27 (13%) identified a cost of phenotypic plasticity in plants (collated in van Kleunen and Fischer 2005). Extending beyond plants, the majority, 62%, of Auld et al.'s (2010) multispecies dataset of 227 pairs of cost estimates in two environments were nonsignificant. These “missing” costs may be due to the good condition (such as being exceptional at resource acquisition) of the organism masking the energetic or resource trade‐offs associated with plasticity (de Lisle and Rowe 2023). Costs may also be masked by environment‐specific conditions, rather than the condition of the organism, according to additional modeling by de Lisle and Rowe (2023). In our study, favorable soil conditions may have masked plasticity costs by alleviating resource limitations. A cost for plasticity in flowering days has been shown under different temperature environments in *A. thaliana* (Stinchcombe et al. 2004). This suggests that certain environmental stressors, such as temperature extremes, can expose the costs of plasticity. Several studies support the idea that costs are more detectable under resource limitations, where compensation for plastic responses is limited (collated in van Kleunen and Fischer 2005). However, Dorn et al. (2000) successfully detected costs in stressful and benign environments for *A. thaliana* . A review of 24 studies identified that the majority of costs that have been detected are environment‐specific (Auld et al. 2010). In our system, identifying such costs may require experimental conditions with stronger environmental contrasts to isolate plasticity effects from background variation in environmental conditions (de Lisle and Rowe 2023).

## 5 Conclusions

Our findings underscore the importance of examining phenotypic plasticity across different trait categories and environmental contexts to better understand the circumstances under which plasticity is adaptive, neutral, or maladaptive. These outcomes have important implications for the evolutionary trajectory of plasticity: whether it is maintained because it is adaptive, can be leveraged as a stepping stone to adaptation and genetically assimilated, or is lost due to being maladaptive. Our results suggest that plasticity can also persist in populations because it is neutral. Future syntheses that distinguish neutral plasticity from maladaptive responses would help clarify the prevalence of this phenomenon and its evolutionary significance.

## Author Contributions

**Maya L. Shamsid‐Deen:** conceptualization (equal), data curation (lead), formal analysis (lead), funding acquisition (equal), investigation (lead), methodology (equal), project administration (equal), software (lead), validation (lead), visualization (lead), writing – original draft (lead), writing – review and editing (lead). **Kenneth D. Whitney:** conceptualization (equal), funding acquisition (equal), investigation (equal), resources (lead), supervision (equal), writing – review and editing (equal).

## Funding

This work was supported by the Biology Graduate Student Association, University of New Mexico and Department of Biology, University of New Mexico.

## Conflicts of Interest

The authors declare no conflicts of interest.

## Data Availability

The data that support the findings of this study are openly available in Dryad at: https://doi.org/10.5061/dryad.3xsj3txvj.

## Appendix A

**TABLE A1:** AICc‐based model comparisons for the relationship of plasticity in seed output and average trait plasticity under fertilizer and competition treatments. Bolded models represent the models utilized for the study. Model statistics include K (number of parameters), AICc, ΔAICc, AICc weight, and log‐likelihood.

Model	K	AICc	ΔAICc	AICc weight	Log‐likelihood
Competition					
Gaussian models (log‐transformed response)					
**Linear**	**3**	**102.86**	**3.23**	**0.11**	**−4.72**
Cubic	5	100.7	1.08	0.33	−43.35
Quadratic	4	99.63	0	0.56	−44.56
Fertilizer					
Gaussian models (square root transformed response)					
Linear	3	105.2	0	0.53	−48.89
Cubic	5	109.22	4.03	0.07	−47.61
Quadratic	4	105.73	0.53	0.4	−47.62
Generalized linear models (raw response)					
**Gamma**	**3**	**19654.79**	**0**	**1**	**−94.69**
Gaussian	3	215.69	18.89	0	−104.14

**TABLE A2:** AICc‐based model comparisons for the relationship of seed output and average trait plasticity under fertilizer and competition treatments. Bolded models represent the models utilized for the study. Model statistics include K (number of parameters), AICc, ΔAICc, AICc weight, and log‐likelihood.

Model	K	AICc	ΔAICc	AICc weight	Log‐likelihood
Competition					
Gaussian models (log‐transformed response)					
**Linear**	**3**	**71.83**	**3.61**	**0.11**	**−32.21**
Cubic	5	70.08	1.86	0.25	−28.04
Quadratic	4	68.22	0	0.64	−28.86
Fertilizer					
Gaussian models (square root transformed response)					
**Linear**	**3**	**211.94**	**0**	**0.45**	**−102.22**
Cubic	5	213.24	1.31	0.23	−99.48
Quadratic	4	212.60	0.66	0.32	−100.97

**TABLE A3:** Results of linear model regressing *plasticity in seed output on trait plasticity* for plants that experienced the competition environment. Coefficients (*β*) are presented on the log scale with 95% confidence intervals, *t*‐values, and *p* values.

Predictor	Estimate	95% CI	*t* (df)	*p*
Trait plasticity (competition)	−0.054	−0.339, 0.231	−0.396 (20)	0.696

**TABLE A4:** Gamma generalized linear model (log link) evaluating the association between average trait plasticity and seed output plasticity under fertilizer treatment. Coefficients (*β*) are presented on the log scale with 95% confidence intervals, *t*‐values, and *p* values.

Predictor	Estimate	95% CI	*t* (df)	*p*
Trait plasticity (fertilizer)	0.003	−0.007, 0.016	0.713 (20)	0.485

**TABLE A5:** Regression table of average plasticity and seed output for each population. Average trait plasticity in the competition environments was log‐transformed, and in the fertilizer environments our response variable was square root transformed.

Predictor	Estimate	95% CI	*t* (df)	*p*
Competition environments				
Trait plasticity (competition)	−0.041	−0.177, 0.095	−0.636	0.532
Fertilizer environments				
Trait plasticity (fertilizer)	0.110	−0.264, 0.485	0.619	0.544

**TABLE A6:** Spearman trait phenotypic plasticity correlation table for the competition environments. Bold indicates statistical significance.

Plasticity trait	Plasticity trait	*r_s_*	Adjusted *p*
Rosette diameter at 1 month	Average plasticity	0.621	0.120
**Rosette diameter at 2 months**	**Average plasticity**	**0.714**	**0.012**
Days vegetative	Average plasticity	0.278	1.000
Leaf count at 1 month	Average plasticity	0.466	1.000
Days flowering	Average plasticity	0.162	1.000
Longevity	Average plasticity	0.395	1.000
Maximum number of siliques	Average plasticity	0.325	1.000
Maximum height	Average plasticity	0.325	1.000
Inflorescence number	Average plasticity	0.261	1.000
Rosette diameter at 1 month	Rosette diameter at 2 months	0.552	0.341
Rosette diameter at 1 month	Days vegetative	0.264	1.000
Rosette diameter at 2 months	Days vegetative	0.144	1.000
**Rosette diameter at 1 month**	**Leaf count at 1 month**	**0.730**	**0.008**
Rosette diameter at 2 months	Leaf count at 1 month	0.319	1.000
Days vegetative	Leaf count at 1 month	0.181	1.000
Rosette diameter at 1 month	Days flowering	−0.164	1.000
Rosette diameter at 2 months	Days flowering	−0.344	1.000
Days vegetative	Days flowering	−0.191	1.000
Leaf count at 1 month	Days flowering	−0.096	1.000
Rosette diameter at 1 month	Longevity	−0.027	1.000
Rosette diameter at 2 months	Longevity	−0.155	1.000
Days vegetative	Longevity	−0.101	1.000
Leaf count at 1 month	Longevity	−0.003	1.000
**Days flowering**	**Longevity**	**0.813**	**< 0.001**
Rosette diameter at 1 month	Maximum number of siliques	0.125	1.000
Rosette diameter at 2 months	Maximum number of siliques	0.143	1.000
Days vegetative	Maximum number of siliques	0.079	1.000
Leaf count at 1 month	Maximum number of siliques	0.065	1.000
Days flowering	Maximum number of siliques	0.009	1.000
Longevity	Maximum number of siliques	0.300	1.000
Rosette diameter at 1 month	Maximum height	0.140	1.000
Rosette diameter at 2 months	Maximum height	0.234	1.000
Days vegetative	Maximum height	−0.116	1.000
Leaf count at 1 month	Maximum height	0.004	1.000
Days flowering	Maximum height	0.004	1.000
Longevity	Maximum height	−0.045	1.000
Maximum number of siliques	Maximum height	0.104	1.000
Rosette diameter at 1 month	Inflorescence number	−0.042	1.000
Rosette diameter at 2 months	Inflorescence number	0.000	1.000
Days vegetative	Inflorescence number	−0.003	1.000
Leaf count at 1 month	Inflorescence number	0.026	1.000
Days flowering	Inflorescence number	0.248	1.000
Longevity	Inflorescence number	0.286	1.000
Maximum number of siliques	Inflorescence number	0.234	1.000
Maximum height	Inflorescence number	0.255	1.000

**TABLE A7:** Spearman trait phenotypic plasticity correlation table for the fertilizer environments. Bold indicates statistical significance.

Plasticity trait	Plasticity trait	*r_s_*	Adjusted *p*
**Rosette diameter at 1 month**	**Average plasticity**	**0.6974**	**0.020**
**Rosette diameter at 2 months**	**Average plasticity**	**0.9052**	**< 0.001**
Days vegetative	Average plasticity	0.1961	1.000
Leaf count at 1 month	Average plasticity	0.5649	0.343
Days flowering	Average plasticity	0.1481	1.000
Longevity	Average plasticity	0.1922	1.000
Maximum number of siliques	Average plasticity	0.4065	1.000
Maximum height	Average plasticity	0.1558	1.000
Inflorescence number	Average plasticity	0.2571	1.000
Rosette diameter at 1 month	Rosette diameter at 2 months	0.599	0.149
Rosette diameter at 1 month	Days vegetative	0.334	1.000
Rosette diameter at 2 months	Days vegetative	0.356	1.000
Rosette diameter at 1 month	Leaf count at 1 month	0.495	0.813
Rosette diameter at 2 months	Leaf count at 1 month	0.604	0.135
Days vegetative	Leaf count at 1 month	0.395	1.000
Rosette diameter at 1 month	Days flowering	−0.138	1.000
Rosette diameter at 2 months	Days flowering	0.230	1.000
Days vegetative	Days flowering	−0.203	1.000
Leaf count at 1 month	Days flowering	0.157	1.000
Rosette diameter at 1 month	Longevity	0.130	1.000
Rosette diameter at 2 months	Longevity	0.403	1.000
Days vegetative	Longevity	0.606	0.128
Leaf count at 1 month	Longevity	0.413	1.000
Days flowering	Longevity	0.148	1.000
Rosette diameter at 1 month	Maximum number of siliques	−0.051	1.000
Rosette diameter at 2 months	Maximum number of siliques	0.216	1.000
Days vegetative	Maximum number of siliques	−0.613	0.113
Leaf count at 1 month	Maximum number of siliques	−0.231	1.000
Days flowering	Maximum number of siliques	0.126	1.000
Longevity	Maximum number of siliques	−0.338	1.000
Rosette diameter at 1 month	Maximum height	−0.139	1.000
Rosette diameter at 2 months	Maximum height	−0.132	1.000
**Days vegetative**	**Maximum height**	**−0.729**	**0.008**
Leaf count at 1 month	Maximum height	−0.336	1.000
Days flowering	Maximum height	0.109	1.000
**Longevity**	**Maximum height**	**−0.770**	**0.002**
**Maximum number of siliques**	**Maximum height**	**0.775**	**0.002**
Rosette diameter at 1 month	Inflorescence number	0.104	1.000
Rosette diameter at 2 months	Inflorescence number	0.127	1.000
Days vegetative	Inflorescence number	−0.264	1.000
Leaf count at 1 month	Inflorescence number	−0.271	1.000
Days flowering	Inflorescence number	−0.053	1.000
Longevity	Inflorescence number	−0.503	0.728
Maximum number of siliques	Inflorescence number	0.361	1.000
Maximum height	Inflorescence number	0.496	0.798
